# Determinants of COVID‐19 vaccine uptake among healthcare professionals and the general population in Cyprus: A web‐based cross‐sectional survey

**DOI:** 10.1111/jep.13764

**Published:** 2022-09-17

**Authors:** Konstantinos Giannakou, Georgia Fakonti, Maria Kyprianidou

**Affiliations:** ^1^ Department of Health Sciences, School of Sciences European University Cyprus Nicosia Cyprus

**Keywords:** COVID‐19, Cyprus, healthcare professionals, SARS‑CoV‑2, vaccination coverage, vaccination uptake

## Abstract

**Objective:**

This study aims to examine the factors influencing COVID‐19 vaccine uptake among healthcare professionals (HCPs) and the general population in Cyprus.

**Methods:**

A web‐based cross‐sectional study was conducted (November 2021–January 2022), using a self‐administered, anonymous questionnaire to collect information covering a wide range of potential determinants including sociodemographic and health‐related characteristics, trust in the healthcare system, satisfaction with it, utilization of preventive healthcare services, COVID‐19 vaccination information and general vaccination knowledge.

**Results:**

A total of 2582 participants completed the survey. Overall, 53.5% of participants representing the general population, and 70.0% of the HCPs received the COVID‐19 vaccination. We found that as the age increases by 1 year among the general population, the odds of being vaccinated against COVID‐19 increase by 1.02 units (95% 1.00, 1.03, *p*= 0.035). In addition, participants among the general population with increased trust in national healthcare authorities' guidelines (OR = 3.96, 95% CI: 3.41, 4.61), and increased vaccination knowledge scores (OR = 1.11, 95% CI: 1.05, 1.18) were significantly more likely to be vaccinated, while those who had underage children living in the household were significantly less likely to be vaccinated against COVID‐19 (OR = 0.68, 95% CI: 0.50, 0.91). Furthermore, male HCPs (OR = 1.91, 95% CI: 1.01, 3.59), and those who reported increased trust in national healthcare authorities' guidelines (OR = 5.38, 95% CI: 3.65, 7.95) were significantly more likely to be vaccinated.

**Conclusion:**

Public health policymakers can use national campaigns and long‐term planning to build public trust in national healthcare authorities and raise awareness about the benefits of vaccination. Such strategies could pave the way for adequate vaccine uptake and prepare the public for unfavourable scenarios, such as future pandemics.

## 1 INTRODUCTION

The severe acute respiratory syndrome coronavirus 2 (SARS‑CoV‑2) was firstly detected in 2019 and is responsible for the coronavirus disease 2019 (COVID‐19). Since its appearance, SARS‐CoV‐2 spread worldwide, and the World Health Organization declared the first coronavirus pandemic in human history in March 2020.^1^ By March 2022 more than 470,000,000 humans have been infected by SARS‐CoV‐2 and approximately 6,000,000 lost their life.^2^ Most people with COVID‐19 experienced mild to moderate symptoms, but some developed life‐threatening illnesses and post‐COVID‐19 conditions with long‐term effects.^3^ Beyond the impact of COVID‐19 on individuals' physical health, the COVID‐19 pandemic also affected their mental health and social interaction.^4^, ^5^

Several vaccines against COVID‐19 have been approved and distributed around the globe; however, vaccine hesitancy remains a crucial public health challenge.^6^ Vaccine hesitancy refers to refusal or delayed acceptance of vaccines, hence beset herd immunity achievement.^7^ The process of vaccine acceptance is a multidimensional and context‐specific topic that varies depending on the specific vaccine, time and place.^7^, ^8^ Different levels of COVID‐19 vaccine acceptance have been reported across the world, with a large proportion of healthcare professionals (HCPs) and public members refusing the COVID‐19 vaccine in some countries.^6^, ^9^, ^10^ Among the reasons for COVID‐19 vaccination refusal were the fear of possible side effects, and concerns over the safety and efficacy of the vaccine.^11^

A growing body of literature recognizes the importance of trust in health services and confidence in the importance of vaccines as influential factors for COVID‐19 vaccination acceptance. Individual's confidence in the usefulness of vaccines, accurate information providence, trust in doctors and the healthcare system, discussions with medical staff about the vaccine importance and previous medical records such as uptake of influenza vaccine are factors that are associated with the COVID‐19 vaccination acceptance.^12^, ^13^, ^14^ Furthermore, the level of misinformation about the usefulness of vaccines, which is often linked with a lack of trust in science is negatively associated with the COVID‐19 vaccination acceptance.^15^, ^16^, ^17^

Professionals in the healthcare sector may have different vaccination knowledge levels, trust in healthcare services and generally different determinants for COVID‐19 vaccination acceptance compared to the general population. However, evidence is scarce on the different determinants that influence HCPs and the general population to get vaccinated against COVID‐19. In Cyprus, studies have identified a low COVID‐19 vaccination acceptance among HCPs with vaccination knowledge being an associated factor for COVID‐19 vaccine acceptance.^18^, ^19^, ^20^ Also, inadequate COVID‐19 vaccine uptake among the general population of Cyprus was recently identified (54% of 2117 participants).^21^ It is critical to gain a comprehensive understanding of the factors that influence vaccination uptake so that future advertising campaigns and government interventions can be more effectively targeted. Therefore, this study aims to examine the factors influencing COVID‐19 vaccine uptake among the HCPs and the general population in Cyprus.

## 2 METHODS

### 2.1 Study design, participants and data collection

This study was reported following the Strengthening the Reporting of Observational Studies in Epidemiology.^22^ This was a web‐based cross‐sectional survey performed between the 15th of November 2021 and the 7th of January 2022. The referent population included Greek‐Cypriot, aged 18 years old and above, living in the five government‐controlled municipalities of the Republic of Cyprus (Nicosia, Limassol, Larnaca, Paphos and Ammochostos). A nonprobability convenience sampling approach was used to recruit participants using an online self‐administered questionnaire, created in Google Forms, and dispersed using instant messaging apps (e.g., WhatsApp, Viber), social media platforms (e.g., Facebook, Instagram), and social networking sites (e.g., LinkedIn). Additional methodological details of this study have been presented elsewhere.^21^, ^23^

### 2.2 Survey instrument

The online questionnaire included 47 open‐ended and closed‐ended questions in the Greek language covering a wide range of potential determinants, including sociodemographic characteristics, health‐related status, information about trust in the healthcare system, use of preventive healthcare services, information about COVID‐19 vaccination, sources of vaccine information and participants' general vaccine knowledge. The questionnaire was developed by the research team, based on previous research experience and extensive literature search.^24^, ^25^, ^26^, ^27^, ^28^, ^29^, ^30^ Face validity was assessed in a pilot study of 50 participants before the actual study to assess the clarity and application of all survey items, as well as to address wording issues. Cronbach's alpha coefficient for internal reliability for the section regarding the attitudes towards healthcare services section was 0.68, while for vaccination knowledge items were 0.71.

### 2.3 Ethics approval

This study was approved by the Cyprus National Bioethics Committee (CNBC) (ΕΕΒΚ ΕΠ 2021.01.219). All participants were informed about the research aims and objectives before taking part and that all data would be used only for research purposes. Participation was completely anonymous and voluntary. Participants consent to take part in research before completing the online questionnaire by answering a “Yes/No” question on a mandatory electronic form.

### 2.4 Statistical analysis

Shapiro–Wilk normality test was applied to examine the normality of the continuous variables. Participants' characteristics are presented as mean ± SD for continuous measures with normal distribution, while continuous characteristics with skewed distributions are presented as median with interquartile range (IQR). Absolute (*n*) and relative (%) frequencies were used to present categorical variables (i.e., gender, geographical area). In the analysis, we divide our study population into two groups: participants representing the general population of Cyprus and HCPs. To assess the association between COVID‐19 vaccination status and the categorical characteristics among participants representing the general population and HCPs separately as well as between vaccinated participants in the general population and vaccinated HCPs, the *χ* ^2^ test of independence was used. The Student's *t*‐test was used for the comparison of the COVID‐19 vaccination status and continuous baseline characteristics with normal distribution among the sample of the general population and HCPs separately as well as between vaccinated participants in the general population and vaccinated HCPs.

Participants' vaccination knowledge was measured using a 12‐item scale. A knowledge score was created for each participant by scoring the individual knowledge question items, giving a score of 1 for each question correctly answered and 0 for each question incorrectly answered or in “I do not know” responses. The knowledge score was calculated by adding the points of each of the 12 knowledge items (maximum score of 12). We have calculated and used the tertiles of the actual data set dividing the sample into equal size subgroups. The tertiles of vaccination knowledge score were defined as follows: low vaccination knowledge (score ≤ 6), moderate vaccination knowledge score (score 7–8) and high vaccination knowledge score (score ≥ 9). Higher scores indicate a higher vaccination knowledge.

Hierarchical logistic regression analysis was used to examine the association between sociodemographic characteristics, presence of chronic diseases, attitudes towards healthcare services and vaccination knowledge score on vaccination status among participants in the general population and HCPs separately. Firstly, we added the sociodemographic characteristics (gender, age, geographical area, marital status, underage children living in the household, education and annual income) as independent variables in a model with vaccination status (Yes vs. No) as a dependent variable (Model 1). Then, we added the presence of chronic diseases, the use of preventive healthcare services, trust in official guidelines, satisfaction with the healthcare system and following doctor's instructions as independent variables (Model 2). Finally, the vaccination knowledge score was added to the model (Model 3). Bar charts were constructed to present the sources of vaccination‐related information. All statistical tests performed were two‐sided with the statistical significance level set at *α* = 0.05. Statistical analysis was conducted using STATA 14.0 (Stata Corp.) and Microsoft Excel 2013.

## 3 RESULTS

### 3.1 Participants' characteristics

A total of 2582 individuals participated in the study. The mean age of the participants was 38.0 years old (SD = 10.5 years old) with 9% (*n* = 231) aged 18–24 years old, 76.9% (*n* = 1986) aged 25–49 years old, 10.0% (*n* = 258) aged 50–59 years old and 4.1% (*n* = 107) aged 60 years and over. Most of the respondents were females (*n* = 1606, 62.2%), from the capital of Cyprus, Nicosia (*n* = 1198, 46.4%) and were married/in cohabitation (*n* = 1792, 70.4%). Approximately half of the participants had underage children living in the household (*n* = 1372, 53.2%), had completed an undergraduate education (*n* = 1186, 46.5%) and had an annual income of more than €19,500 (*n* = 1238, 49.2%) (Table 1). There was a total of 504 HCPs in the study among whom 223 (48%) were nursing staff, 76 (16.3%) were pharmacists, 73 (15.7%) were physicians, 62 (13.3%) were other nonmedical professionals (i.e., laboratory workers, nutritionists, occupational therapists, psychologists, radiologists, speech and language therapists, care assistants and administrative personnel) and 31 (6.7%) were physiotherapists.

**Table 1:** Sociodemographic characteristics of all participants, overall and by vaccination status

Characteristics	Overall (*N* = 2582)	COVID‐19 vaccination by general population			COVID‐19 vaccination by HCPs		
		No (*N* = 956, 46.5%)	Yes (*N* = 1099, 53.5%)	*p* Value	No (*N* = 147, 30.0%)	Yes (*N* = 350, 70.0%)	*p* Value
Gender [*N*,^a^ %]							
Female	1606 (62.2)	579 (45.4)	696 (54.6)	0.198^b^	97 (30.7)	219 (69.3)	0.470^c^
Male	976 (37.8)	377 (48.3)	403 (51.7)		50 (27.6)	131 (72.4)	
Age	38.0 ± 10.5^d^	37.6 ± 9.8^d^	38.9 ± 11.4^d^	**0.007** e	33 (29–41)^f^	36 (30–44)^f^	0.259^g^
Age group (*N*,^a^ %)							
18–24	231 (9.0)	93 (46.0)	109 (54.0)	**<0.001** ^b^	8 (30.8)	18 (69.2)	0.959^c^
25–49	1986 (76.9)	748 (48.2)	803 (51.8)		123 (29.9)	288 (70.1)	
50–59	258 (10.0)	92 (44.2)	116 (55.8)		13 (27.1)	35 (72.9)	
>60	107 (4.1)	23 (24.5)	71 (75.5)		3 (25.0)	9 (75.0)	
Geographical area (*N*,^a^ %)							
Nicosia	1198 (46.4)	416 (43.4)	542 (56.6)	**0.008** ^b^	67 (29.4)	161 (70.6)	0.422^c^
Limassol	625 (24.2)	272 (52.1)	250 (47.9)		30 (31.3)	66 (68.7)	
Larnaca	392 (15.2)	132 (43.4)	172 (56.6)		21 (25.0)	63 (75.0)	
Paphos	174 (6.7)	62 (47.3)	69 (52.7)		9 (23.7)	29 (76.3)	
Ammochostos	193 (7.5)	74 (52.9)	66 (47.1)		20 (39.2)	31 (60.8)	
Marital status, (*N*,^h^ %)							
Married/In cohabitation	1792 (70.4)	652 (46.0)	765 (54.0)	0.313^b^	98 (27.2)	263 (72.8)	0.202^c^
Unmarried	583 (22.9)	215 (45.6)	257 (54.4)		34 (33.0)	69 (67.0)	
Divorced/separated/widowed	172 (6.7)	75 (52.5)	68 (47.5)		11 (40.7)	16 (59.3)	
Underage children living in the household (*N* i, %)							
No	1209 (46.8)	400 (41.7)	558 (58.3)	**<0.001** ^b^	76 (32.8)	156 (67.2)	0.146^c^
Yes	1372 (53.2)	556 (50.7)	540 (49.3)		71 (26.8)	194 (73.2)	
Education (*N*,^j^ %)							
Up to secondary education	415 (16.3)	208 (51.0)	200 (49.0)	**<0.001** ^b^	0 (0.0)	0 (0.0)	0.127^c^
Undergraduate education	1186 (46.5)	458 (49.4)	469 (50.6)		80 (32.4)	167 (67.6)	
Postgraduate education	951 (37.2)	276 (39.5)	423 (60.5)		64 (26.1)	181 (73.9)	
Annual income (*N*,^k^ %)							
Low (≤€6500)	293 (11.7)	134 (46.7)	153 (53.3)	**0.001** ^b^	0 (0.0)	5 (100.0)	**0.006** ^c^
Moderate (€6500–19,500)	982 (39.1)	413 (51.0)	397 (49.0)		60 (37.7)	99 (62.3)	
High (>€19,500)	1238 (49.2)	377 (41.8)	525 (58.2)		82 (25.2)	244 (74.8)	

Abbreviation: HCPs, healthcare professionals.

^a^ *N* = 2582.

^b^ Differences between COVID‐19 vaccination groups among the general population were tested using chi‐square test.

^c^ Differences between COVID‐19 vaccination groups among HCPs were tested using chi‐square test.

^d^ Age is presented as mean ± SD.

^e^ Differences between COVID‐19 vaccination groups among general population were tested using *t*‐test.

^f^ Age is presented as median (interquartile range).

^g^ Differences between COVID‐19 vaccination groups among HCPs were tested using Kolmogorov–Smirnov test.

^h^ *N* = 2547.

^i^ *N* = 2581.

^j^ *N* = 2552.

^k^ *N* = 2513.

### 3.2 Determinants of vaccination status by sociodemographic characteristics, health status, attitude towards healthcare services, and knowledge about COVID‐19

Overall, we found that 1449 (56.1%) of the respondents were vaccinated against COVID‐19. Among the participants representing the general population of Cyprus, we reported 1099 (53.5%) vaccinated participants. Vaccinated individuals were significantly older (mean=38.9 years old, SD = 11.4) compared to the unvaccinated individuals (mean = 37.6 years old, SD = 9.8) (*p* = 0.007). More than half of the residents in all geographical areas of Cyprus were vaccinated, except for Limassol (*n* = 250, 47.9%) and Ammochostos (*n* = 66, 47.1%) (*p* = 0.008; Table 1).

Specific sociodemographic characteristics of participants representing the general population of Cyprus were associated with their COVID‐19 vaccination status. Many participants in the general population who had underage children living in the household were unvaccinated (*n* = 556, 50.7%), while most of the participants without underage children were vaccinated (*n* = 558, 58.3%) (*p* < 0.001). The largest statistically significant differences between vaccination status groups were reported among those who had completed a postgraduate education (39.5% vs. 60.5%, for no and yes, respectively, *p* < 0.001) and participants who had an annual income of more than 19,500 euros (41.8% vs. 58.2%, for no and yes, respectively, *p* < 0.001) (Table 1).

As regards the COVID‐19 vaccination by HCPs, we reported that 350 (70.0%) were vaccinated against COVID‐19. A statistically significant difference was identified among HCPs who had an annual income of more than 19,500 euros (25.2% vs. 74.8%, for no and yes, respectively, *p* = 0.006) (Table 1).

Around 19% (*n* = 482) of the participants reported at least one chronic disease, while most of the participants used preventive healthcare services (*n* = 874, 34.0%) moderately. We also reported that a similar number of participants had no trust (*n*= 680, 26.5%) or a strong trust (*n* = 693, 27.0%) in the official guidelines and recommendations of the national healthcare authorities. Moreover, many participants were moderately satisfied with the healthcare system (*n* = 987, 38.4%) and more than half often follow doctor's instructions (*n* = 1330, 51.7%) (Table 2).

**Table 2:** Information about participants' health status and attitudes towards healthcare services, overall and by vaccination status

Healthcare services' information and attitudes	Overall (*N* = 2582)	COVID‐19 vaccination by general population			COVID‐19 vaccination by HCPs		
		No (*N* = 956, 46.5%)	Yes (*N* = 1099, 53.5%)	*p* Value^a^	No (*N* = 147, 30.0%)	Yes (*N* = 350, 70.0%)	*p* value^b^
Chronic diseases (at least one) (*N*,^c^ %)							
No	2088 (81.3)	808 (48.4)	863 (51.6)	**<0.001**	122 (31.0)	372 (69.0)	0.107
Yes	482 (18.7)	142 (37.6)	236 (62.4)		23 (22.8)	78 (77.2)	
Use of preventive healthcare services (e.g., annual check‐up) (*N*,^d^ %)							
Not at all	198 (7.7)	106 (61.6)	66 (38.4)	**<0.001**	7 (30.4)	16 (69.6)	0.395
Little	674 (26.2)	298 (54.3)	251 (45.7)		43 (35.5)	78 (64.5)	
Moderate	874 (34.0)	300 (44.0)	381 (56.0)		45 (24.9)	136 (75.1)	
Often	689 (26.8)	212 (39.3)	327 (60.7)		44 (30.3)	101 (69.7)	
Very often	136 (5.3)	37 (33.9)	72 (66.1)		7 (28.0)	18 (72.0)	
Trust in official guidelines and recommendations by the national healthcare authorities (*N*,^e^ %)							
No trust	680 (26.5)	550 (91.2)	53 (8.8)	**<0.001**	65 (87.8)	9 (12.2)	**<0.001**
Little trust	307 (12.0)	172 (66.9)	85 (33.1)		28 (62.2)	17 (37.8)	
Moderate trust	606 (23.5)	182 (38.5)	291 (61.5)		44 (35.5)	80 (64.5)	
Strong trust	693 (27.0)	40 (7.8)	472 (92.2)		9 (5.1)	169 (94.9)	
Very strong trust	283 (11.0)	4 (2.0)	198 (98.0)		0 (0.0)	75 (100.0)	
Satisfaction with the healthcare system (*N*,^d^ %)							
No satisfied	466 (18.1)	325 (80.6)	78 (19.4)	**<0.001**	44 (75.9)	14 (24.1)	**<0.001**
Little satisfied	555 (21.6)	303 (63.7)	173 (36.3)		37 (50.0)	37 (50.0)	
Moderate satisfied	987 (38.4)	270 (35.8)	484 (64.2)		50 (22.1)	176 (77.9)	
Very satisfied	510 (19.8)	49 (13.0)	328 (87.0)		15 (12.0)	110 (88.0)	
Extremely satisfied	53 (2.1)	5 (12.8)	34 (87.2)		0 (0.0)	13 (100.0)	
Following doctor's instructions/Medical adherence (*N*,^f^ %)							
Not at all	25 (1.0)	16 (84.2)	3 (15.8)	**<0.001**	4 (66.7)	2 (33.3)	**<0.001**
Little	129 (5.0)	106 (91.4)	10 (8.6)		6 (54.5)	5 (45.5)	
Moderate	429 (16.7)	240 (67.6)	115 (32.4)		36 (50.0)	36 (50.0)	
Often	1330 (51.7)	432 (41.2)	616 (58.8)		73 (27.5)	192 (72.5)	
Very often	660 (25.6)	158 (30.9)	354 (69.1)		27 (19.2)	114 (80.8)	

*Note*: Bold values indicate statistically significant associations (*p* < 0.05).

Abbreviation: HCPs; healthcare professionals.

^a^ Differences between COVID‐19 vaccination groups among general population were tested using chi‐square test.

^b^ Differences between COVID‐19 vaccination groups among HCPs were tested using chi‐square test.

^c^ *N* = 2570.

^d^ *N* = 2571.

^e^ *N* = 2569.

^f^ *N* = 2573.

Of note, most of the participants representing the general population of Cyprus with at least one chronic disease were vaccinated against COVID‐19 (*n* = 236, 62.4%). We reported the largest differences in the usage of preventive healthcare services among those who very often use them (33.9% vs. 66.1%, for no and yes, respectively) or do not use them at all (61.5% vs. 38.5%, for no and yes, respectively) (*p* < 0.001). The largest differences between the vaccination status groups were observed among the sample of the general population who had very strong trust in official guidelines and recommendations by the national healthcare authorities (2.0% vs. 98.0%, for no and yes, respectively), who are extremely satisfied with the healthcare system (12.8% vs. 87.2%, for no and yes, respectively) and those who follow doctors' instructions very often (30.9% vs. 69.1%, for no and yes, respectively) (*p* < 0.001). We also reported that all HCPs who have a very strong trust in official guidelines and recommendations by the national healthcare authorities, and those who are extremely satisfied with the healthcare system were vaccinated against COVID‐19 (*p* < 0.001). Interestingly, the largest difference between HCPs' vaccination status groups was found in those who follow doctor's instructions very often (19.2% vs. 80.8%, for no and yes, respectively) (*p* < 0.001) (Table 2).

Among the vaccinated participants, 6.1% (*n* = 88) received only one dose of the COVID‐19 vaccine, while 62.7% (*n* = 908) and 31.2% (*n* = 452) received two and three doses respectively. Most of the participants had an intention to receive another dose if requested (*n* = 654, 45.2%), and they believe that the vaccine very much helped to prevent the development of COVID‐19 (*n* = 459, 31.8%; Supporting Information: Supplementary Table 1).

The participants' mean vaccination knowledge score was 6.9 (SD = 2.6) which indicates a low to moderate vaccination knowledge. Specifically, the majority have a low vaccination knowledge level (*n* = 1082, 41.9%), followed by a high (*n* = 779, 30.2%) and a moderate (*n* = 721, 27.9%) vaccination knowledge level. Among the sample of the general population of Cyprus, the vaccinated individuals had a higher vaccination knowledge score (mean = 7.4, SD = 2.2) compared to the unvaccinated group (mean = 5.4, SD = 2.4) (*p* < 0.001). Regarding HCPs' vaccination knowledge score, we found that vaccinated HCPs had a higher vaccination knowledge score (mean = 9.0, SD = 1.7) compared to unvaccinated HCPs (mean = 7.5, SD = 2.2; *p* < 0.001; Table 3).

**Table 3:** Participants' general vaccination knowledge, overall and by vaccination support

		COVID‐19 vaccination by general population			COVID‐19 vaccination by HCPs		
	Overall (*N* = 2582)	No (*N* = 956, 46.5%)	Yes (*N* = 1099, 53.5%)	*p* Value	No (*N* = 147)	Yes (*N* = 350)	*p* Value
Mean knowledge score^a^ (SD)	6.9 ± 2.6	5.4 ± 2.4	7.4 ± 2.2	**<0.001** b	7.5 ± 2.2	9.0 ± 1.7	**<0.001** c
Knowledge level (*N*,^d^ %)							
Low (score ≤ 6)	1082 (41.9)	644 (64.5)	355 (35.5)	**<0.001** e	42 (62.7)	25 (37.3)	**<0.001** f
Moderate (score 7–8)	721 (27.9)	212 (36.2)	374 (63.8)		47 (37.0)	80 (63.0)	
High (score ≥ 9)	779 (30.2)	100 (21.3)	370 (78.7)		58 (19.1)	245 (80.9)	

Abbreviation: HCPs, healthcare professionals.

*Note*: bold values indicate statistically significant associations (*p* < 0.05).

^a^ Range of knowledge score 0–12.

^b^ Differences between COVID‐19 vaccination groups among general population were tested using *t*‐test.

^c^ Differences between COVID‐19 vaccination groups among HCPs were tested using *t*‐test.

^d^ *N* = 2582.

^e^ Differences between COVID‐19 vaccination groups among general population were tested using chi‐square test.

^f^ Differences between COVID‐19 vaccination groups among HCPs were tested using chi‐square test.

To identify independent determinants of COVID‐19 vaccination coverage, hierarchical logistic regression modelling was applied (Supporting Information: Table 2). Firstly, we applied a model adding several sociodemographic characteristics of the participants representing the general population of Cyprus (Model 1). We found that as the age increases by 1 year among the general population, the odds of being vaccinated against COVID‐19 increase by 1.01 units (95% CI: 1.00, 1.02, *p* = 0.022). In addition, residents of Limassol (OR = 0.74, 95% CI: 0.59, 0.92, *p* = 0.008) and those who had underage children living in the household (OR = 0.63, 95% CI: 0.51, 0.77, *p* < 0.001) were significantly less likely to be vaccinated against COVID‐19 compared to residents of Nicosia and those without underage children living in the household, respectively. Furthermore, individuals who completed a postgraduate education were more likely to be vaccinated compared to those who completed up to secondary education (OR = 1.40, 95% CI: 1.06, 1.86, *p* = 0.018). When the presence of chronic diseases and attitudes towards healthcare services were added to the model (Model 2), age (*p* = 0.042) and having underage children living in the household (*p* = 0.010) remained statistically significant. In addition, increased trust in national healthcare authorities' guidelines was associated with vaccination status (OR = 4.26, 95% CI: 3.68, 4.93, *p* < 0.001). Finally, when the vaccination knowledge score was added to the model (Model 3), the association for age (*p* = 0.035), having underage children living in the household (*p* = 0.009), and increased trust in national healthcare authorities' guidelines (*p* < 0.001) with vaccination status remained statistically significant. We also found that for every one unit increase in vaccination knowledge score, the probability of being vaccinated against COVID‐19 increases by 1.11 times (95% CI: 1.05, 1.18, *p* < 0.001).

Regarding the hierarchical logistic regression model for vaccination status among HCPs, we reported a statistically significant association between trust in official guidelines and vaccination status (*p* < 0.001) after adjusting for sociodemographic characteristics, presence of chronic diseases and attitudes towards healthcare services (Model 2). Specifically, increased trust in national healthcare authorities' guidelines increases the probability of being vaccinated against COVID‐19 (OR = 5.71, 95% CI: 3.88, 8.39, *p* < 0.001). When vaccination knowledge score was added to the model (Model 3), the association between increased trust in national healthcare authorities' guidelines (*p* < 0.001) with vaccination status remained statistically significant. In addition, we found that male HCPs were significantly more likely to be vaccinated against COVID‐19 (95% OR = 1.91, CI: 1.01, 3.59, *p* = 0.045) (Supporting Information: Table 2).

### 3.3 Sources of information about vaccination

**Figure 1:**
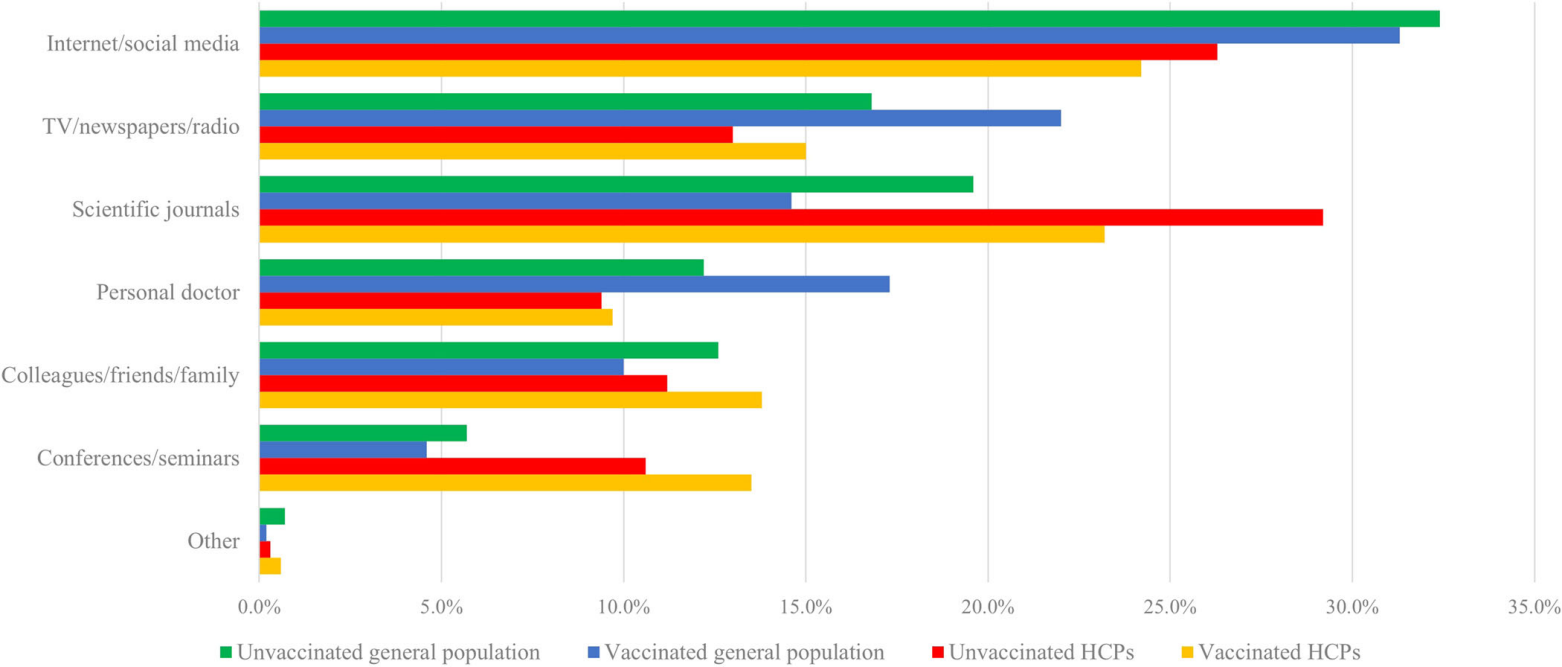
Sources of information about vaccination among the vaccinated and unvaccinated participants representing the general population of Cyprus and HPCs.

Figure 1 presents the main sources of information about vaccination among the vaccinated and unvaccinated participants of general population of Cyprus and HPCs. The majority of the unvaccinated sample representing the general population of Cyprus reported internet/social media (32.4%) and scientific journals (19.6%) as the main sources of information, while among the sample of vaccinated individuals the main sources of information were internet/social media (31.3%) and TV/newspapers/radio (22.0%). Furthermore, among the sample of vaccinated HCPs the main sources of information were internet/social media (24.2%) and scientific journals (23.2%), while the scientific journals (29.2%) and internet/social media (26.3%) were the primary sources of information among unvaccinated HCPs.

## 4 DISCUSSION

This study aimed to investigate the factors influencing COVID‐19 vaccine uptake among the participants of the general population of Cyprus and HCPs to advance our understanding of distinct factors that affect their decision. According to our results, as of 7 January 2022, vaccination coverage against COVID‐19 was 53.5% of the participants representing the general population and 70.0% of HCPs. The strongest determinants of being vaccinated against COVID‐19 among the sample of the general population were older age, underage children residing in the household, increased trust in official healthcare authorities' guidelines, and increased vaccination knowledge. Furthermore, the strongest determinants of being vaccinated against COVID‐19 among the sample of HCPs were being male and increased trust in official healthcare authorities' guidelines.

Our results showed that more than half (53.5%) of the participants representing the general population of Cyprus were vaccinated against COVID‐19 and one of the strongest determinants of vaccine uptake was age. Of note, a similar proportion of vaccinated individuals (54.0%, *n* = 2117) was reported in another study published in March 2022 in Cyprus with age being a significant factor in supporting mandatory COVID‐19 vaccination.^21^ Epidemiological studies also showed that COVID‐19 vaccination is higher in older people in Wales (73% of adults and 92% of those over 50 years old) and the United States (38.3% of 18–29 years old and 80.0% of over 65 years old).^31^, ^32^ In addition, a study among the elderly Swedish general population (aged ≤60 years old) revealed that younger age is a significant factor of lower vaccine uptake.^33^ Although the COVID‐19 vaccination had been started in most countries since December 2020, only a few epidemiological studies investigated the factors influencing COVID‐19 vaccine uptake, while the majority of studies investigated individuals' willingness to receive the COVID‐19 vaccine and age was identified as an influential factor.^34^, ^35^, ^36^, ^37^, ^38^ Specifically, a systematic review that investigated COVID‐19 vaccination intention in the first year of the pandemic found that older individuals were more likely to be vaccinated against COVID‐19, and only a few studies associated younger age groups with increasing willingness.^37^

The link between older age and higher vaccination uptake among the sample of the general population could be explained in different ways. For example, older individuals are more susceptible to COVID‐19 and considered themselves more vulnerable.^39^, ^40^, ^41^ In addition, it is known that the influenza vaccination is recommended for individuals aged ≤65 years old due to a higher risk of disease severity, hospitalization as well as death by influenza infection.^42^ Hence, it is possible that older individuals and those with chronic diseases prefer to be vaccinated against influenza and COVID‐19 too.^43^ It is also important to acknowledge the early availability of COVID‐19 vaccines to older and immunocompromised individuals which can impact the vaccination uptake among the different aged populations. However, other studies found that younger individuals are more likely to accept the COVID‐19 vaccine compared to older individuals.^44^, ^45^, ^46^

We also observed that the presence of underage children in the household was associated with a lower probability of COVID‐19 vaccination uptake. This finding is consistent with other research studies,^40^, ^47^, ^48^ however, the reasons contributing to the observed outcome need further investigation. A possible explanation could be the parental fear of any long‐term health problem caused by the vaccine that will interfere with their ability to raise their children. Supporting this hypothesis, a recent study showed that only a small percentage of mothers in Greece did not have doubts about the efficacy and safety of new vaccines.^49^ The same study also showed that the majority of participants are against the immediate vaccination of children after the release of new vaccines.^49^ In addition, many COVID‐19 vaccines were not approved for young children, so parents may think vaccines were unsafe for their children and themselves.

Our study also found that increased level of general vaccination knowledge was significantly associated with the uptake of the COVID‐19 vaccine, even after adjusting for several sociodemographic characteristics and other factors. Our finding is consistent with other studies which have reported that vaccination knowledge is associated with COVID‐19 vaccine hesitancy.^50^, ^51^, ^52^ Individuals with a lower vaccination knowledge level are more likely to believe in misinformation regarding the safety and the side effects of the vaccines and therefore refuse vaccination.^53^, ^54^, ^55^ Of note, the general vaccination knowledge is not only associated with COVID‐19 vaccine acceptance but also associated with the acceptance of several vaccines (i.e., tetanus and influenza).^56^

Knowledge about health issues is related to health decision‐making ability which includes vaccination acceptance.^57^, ^58^ Therefore, it is important to promote scientifically correct information to improve general population knowledge about the COVID‐19 vaccine, especially through the internet, social media, TV, newspapers, and radio which are the main sources of information about vaccination as our results suggest and had a crucial role in the spread of the information related to COVID‐19 (i.e., cases, hospitalizations, and vaccinations) during the COVID‐19 pandemic.^53^, ^59^ Furthermore, we found that public and HCPs trust in official guidelines and recommendations by the national healthcare authorities was associated with a greater probability of being vaccinated against COVID‐19. Similar to our results, lack of trust in the federal government during the pandemic was associated with a lower probability of delaying or refusing routine and COVID‐19 vaccination among other research studies.^37^, ^47^, ^60^, ^61^, ^62^

This study reveals the determinants of COVID‐19 vaccine uptake in Cyprus and stresses the importance of trust in the healthcare authorities on individuals' decision to be vaccinated against COVID‐19. In addition, highlights the crucial role of vaccination knowledge, and other factors that influence general population to be vaccinated against COVID‐19. Since the COVID‐19 vaccine uptake among the sample of general population in Cyprus is insufficient, public health efforts may focus on educational awareness regarding the benefits of COVID‐19 vaccination. According to our findings, for both HCPs and the sample representing the general population of Cyprus, the COVID‐19 vaccine uptake was associated with the trust in healthcare authorities, therefore, public health policymakers may invest in campaigns and future planning to improve populations' trust in the national healthcare authorities. It is of vital importance to maximize the effectiveness of targeted vaccination campaigns to improve the low COVID‐19 vaccine uptake among the citizens of Cyprus. Implementation of such strategies may lay the groundwork for adequate uptake of vaccines and prepare the public for unfavourable situations such as future pandemics. Transparency about vaccine risks and uncertainties surrounding new vaccines and emerging virus strains may also help to boost public confidence in government institutions.^63^ Finally, further research is needed to understand the impact of trust in government and healthcare authorities on COVID‐19 vaccine uptake.

Notwithstanding our major research findings, several limitations of this study should be discussed. Due to the cross‐sectional design, causal inferences cannot be made. Furthermore, because this research relied on voluntary, self‐reported data, we cannot exclude the possibility of social desirability bias, which is common in surveys. Our study's representativeness may be limited by the participants' recruitment method and data collection strategy, which is characterized by an online convenience sampling strategy. In addition, in comparison to the general population, our sample contains a small proportion of participants over the age of 60, which limits the representativeness of our study. Furthermore, we cannot rule out the possibility that people who do not have access to technology are underrepresented in our sample, while certain sub‐groups may be oversampled, lowering the study's overall reliability. Similarly, because data were collected through multiple online channels, we cannot rule out the possibility of duplicate records and responses outside the target population, limiting the study's representativeness and generalizability. Nonetheless, despite the use of an online non‐probabilistic sampling approach, which is an alternate solution for data gathering during virus outbreaks, we ensured that our sample is nationwide, covering all geographical locations under the Republic of Cyprus's authority. Also, we were unable to calculate the response rate for our web‐based survey because there is no way of knowing how many individuals saw the survey or its links but chose not to participate. Finally, these findings apply solely to the population of Cyprus and cannot be generalized to other countries.

## 5 CONCLUSIONS

To our knowledge, this is the first study that provides insights regarding factors influencing COVID‐19 vaccine uptake among participants representing the general population of Cyprus and HCPs. Older age, underage children residing in the household, increased trust in official healthcare authorities' guidelines, and general vaccination knowledge were found to be the strongest determinants of vaccination among the sample representing the general population, whereas gender male and increased trust in official healthcare authorities' guidelines were found to be the strongest determinants of vaccination among HCPs. National campaigns and long‐term planning can be used by public health policymakers to increase public trust in national healthcare authorities while also raising awareness about the benefits of vaccination. Implementing such strategies could pave the way for adequate vaccine uptake and prepare the public for unfavourable scenarios such as future pandemics.

## AUTHOR CONTRIBUTIONS

Konstantinos Giannakou conceived and designed the survey, collected, and analysed the data, draft the original manuscript, and interpreted the results, and supervised this research study; Georgia Fakonti drafted the original manuscript and interpreted the results; Maria Kyprianidou designed the survey, collected, and analysed the data, and draft the original manuscript and interpreted the results. All the authors take responsibility for all aspects of the reliability and freedom from bias of the data presented and their discussed interpretation. All authors read and approved the final manuscript.

## CONFLICT OF INTEREST

The authors declare no conflict of interest.

## Supporting information

Supporting information.

## Data Availability

The underlying data used in this analysis will be made available upon reasonable request.
