# Supplementary material for: Determinants of COVID‐19 vaccine uptake among healthcare professionals and the general population in Cyprus: A web‐based cross‐sectional survey
Source: J Eval Clin Pract. 2022 Sep 17:10.1111/jep.13764. Online ahead of print. doi: 10.1111/jep.13764 (PMC9538130; doi:10.1111/jep.13764)
Supplement: Supplementary file 1 — Supporting information. [file JEP-9999-0-s001.docx]

**Supplementary Table 1.** Information about participants’ COVID-19 vaccination.

|  | **Overall**  (N=1449) | **Vaccinated general population**  (N=1099) | **Vaccinated HCPs**  (N=350) | **p-value**^1^ |
| --- | --- | --- | --- | --- |
| **Number of doses** [N^a^ (%)] | | | | |
| 1 | 88 (6.1) | 70 (6.4) | 18 (5.2) | **<0.001**^f^ |
| 2 | 908 (62.7) | 732 (66.6) | 176 (50.4) |  |
| 3 | 452 (31.2) | 297 (27.0) | 155 (44.4) |  |
| **Type of COVID-19 vaccine** [N^b^ (%)] | | | | |
| Pfizer | 983 (67.8) | 750 (68.2) | 233 (66.6) | **0.001**^f^ |
| Moderna | 167 (11.5) | 137 (12.5) | 30 (8.6) |  |
| Astra Zeneca | 165 (11.4) | 116 (10.6) | 49 (14.0) |  |
| Johnson & Johnson | 69 (4.8) | 58 (5.3) | 11 (3.1) |  |
| Combination | 65 (4.5) | 35 (3.5) | 27 (7.7) |  |
| **Intention to receive another dose if requested** [N^c^ (%)] | | | | |
| Not at all | 139 (9.6) | 108 (9.8) | 31 (8.9) | 0.882^f^ |
| Little | 92 (6.4) | 72 (6.6) | 20 (5.7) |  |
| Moderate | 185 (12.8) | 138 (12.6) | 47 (13.5) |  |
| A lot | 376 (26.0) | 280 (25.5) | 96 (27.5) |  |
| Very much | 654 (45.2) | 499 (45.5) | 155 (44.4) |  |
| **Belief that vaccine helped to prevent the development of COVID-19** [N^d^ (%)] | | | | |
| Not at all | 106 (7.3) | 88 (8.0) | 18 (5.1) | 0.068^f^ |
| Little | 122 (8.4) | 96 (8.8) | 26 (7.5) |  |
| Moderate | 260 (18.0) | 204 (18.6) | 56 (16.0) |  |
| A lot | 498 (34.5) | 358 (32.7) | 140 (40.0) |  |
| Very much | 459 (31.8) | 349 (31.9) | 110 (31.4) |  |
| **They belong to a vulnerable group (diabetic, immunosuppressed, etc.) to whom vaccination is recommended** [N^e^ (%)] | | | | |
| I do not know | 64 (4.6) | 53 (5.0) | 11 (3.3) | 0.412^f^ |
| No | 1125 (80.6) | 854 (80.4) | 271 (81.1) |  |
| Yes | 207 (14.8) | 155 (14.6) | 52 (15.6) |  |
| Abbreviations: HCPs; healthcare professionals; ^a^N=1448; ^b^N=1449; ^c^N=1446; ^d^N=1445; ^e^N=1396; ^f^Differences between vaccinated general population and vaccinated HCPs were tested using chi^2^ test; Bold values indicate statistically significant associations (p<0.05). | | | | |

**Supplementary Table 2**. Hierarchical logistic regression models for sociodemographic, vaccination knowledge score, and health status and attitudes towards healthcare services on vaccination status.

|  | **COVID-19 vaccination by general population** | | | | | | **COVID-19 vaccination by HCPs** | | | | | |
| --- | --- | --- | --- | --- | --- | --- | --- | --- | --- | --- | --- | --- |
|  | **Model 1^a^** | | **Model 2^b^** | | **Model 3^c^** | | **Model 1^a^** | | **Model 2^b^** | | **Model 3^c^** | |
| **Characteristics** | **OR (95% CI)** | **p-value** | **OR (95% CI)** | **p-value** | **OR (95% CI)** | **p-value** | **OR (95% CI)** | **p-value** | **OR (95% CI)** | **p-value** | **OR (95% CI)** | **p-value** |
| **Gender** | | | | | | | | | | | | |
| Female | *Ref* |  | *Ref* |  | *Ref* |  | *Ref* |  | *Ref* |  | *Ref* |  |
| Male | 0.86 (0.70, 1.04) | 0.113 | 0.90 (0.68, 1.18) | 0.447 | 0.95 (0.72, 1.25) | 0.695 | 1.10 (0.72, 1.69) | 0.660 | 1.87 (1.00, 3.52) | 0.050 | **1.91 (1.01, 3.59)** | **0.045** |
| **Age** | **1.01 (1.00, 1.02)** | **0.022** | **1.02 (1.00, 1.03)** | **0.042** | **1.02 (1.00, 1.03)** | **0.035** | 1.01 (0.99, 1.04) | 0.360 | 1.01 (0.97, 1.05) | 0.655 | 1.01 (0.97, 1.05) | 0.675 |
| **Geographical area** | | | | | | | | | | | | |
| Nicosia | *Ref* |  | *Ref* |  | *Ref* |  | *Ref* |  | *Ref* |  | *Ref* |  |
| Limassol | **0.74 (0.59, 0.92)** | **0.008** | 0.94 (0.68, 1.28) | 0.683 | 0.95 (0.70, 1.30) | 0.761 | 0.86 (0.49, 1.48) | 0.581 | 0.61 (0.27, 1.38) | 0.236 | 0.65 (0.29, 1.49) | 0.311 |
| Larnaca | 1.05 (0.80, 1.38) | 0.719 | 1.40 (0.95, 2.05) | 0.086 | 1.35 (0.92, 1.98) | 0.123 | 1.34 (0.73, 2.45) | 0.348 | 0.98 (0.44, 2.19) | 0.954 | 0.99 (0.44, 2.21) | 0.973 |
| Paphos | 0.95 (0.65, 1.40) | 0.811 | 1.09 (0.63, 1.86) | 0.764 | 1.11 (0.65, 1.92) | 0.696 | 1.37 (0.60, 3.11) | 0.457 | 0.75 (0.17, 3.21) | 0.693 | 0.67 (0.15, 2.92) | 0.595 |
| Ammochostos | 0.81 (0.56, 1.18) | 0.278 | 0.81 (0.48, 1.35) | 0.415 | 0.80 (0.48, 1.34) | 0.400 | 0.72 (0.37, 1.40) | 0.328 | 0.54 (0.22, 1.30) | 0.169 | 0.59 (0.24, 1.44) | 0.247 |
| **Marital status** | | | | | | | | | | | | |
| Married/In cohabitation | *Ref* |  | *Ref* |  | *Ref* |  | *Ref* |  | *Ref* |  | *Ref* |  |
| Unmarried | 0.96 (0.73, 1.26) | 0.775 | 0.91 (0.63, 1.33) | 0.629 | 0.89 (0.61, 1.30) | 0.556 | 1.07 (0.60, 1.91) | 0.814 | 1.10 (0.51, 2.38) | 0.813 | 1.17 (0.54, 2.55) | 0.692 |
| Divorced/separated/widowed | 0.69 (0.48, 0.99) | 0.045 | 0.91 (0.54, 1.52) | 0.722 | 0.91 (0.54, 1.52) | 0.715 | 0.51 (0.21, 1.25) | 0.141 | 0.50 (0.16, 1.57) | 0.235 | 0.58 (0.18, 1.86) | 0.359 |
| **Underage children living in the household** | | | | | | | | | | | | |
| No | *Ref* |  | *Ref* |  | *Ref* |  | *Ref* |  | *Ref* |  | *Ref* |  |
| Yes | **0.63 (0.51, 0.77)** | **<0.001** | **0.68 (0.51, 0.91)** | **0.010** | **0.68 (0.50, 0.91)** | **0.009** | 1.12 (0.71, 1.77) | 0.637 | 1.17 (0.63, 2.17) | 0.618 | 1.13 (0.61, 2.12) | 0.692 |
| **Education** | | | | | | | | | | | | |
| Up to secondary education | *Ref* |  | *Ref* |  | *Ref* |  | -* |  | -* |  | -* |  |
| Undergraduate education | 0.98 (0.76, 1.26) | 0.870 | 1.01 (0.71, 1.44) | 0.934 | 0.97 (0.68, 1.38) | 0.849 | *Ref* |  | *Ref* |  | *Ref* |  |
| Postgraduate education | **1.40 (1.06, 1.86)** | **0.018** | 0.96 (0.65, 1.43) | 0.850 | 0.88 (0.59, 1.32) | 0.533 | 1.19 (0.78, 1.81) | 0.412 | 1.39 (0.77, 2.50) | 0.277 | 1.43 (0.79, 2.59) | 0.238 |
| **Annual income** | | | | | | | | | | | | |
| Low (≤ €6,500) | *Ref* |  | *Ref* |  | *Ref* |  | -* |  | -* |  | -* |  |
| Moderate (€6,500 - 19,500) | 0.83 (0.62, 1.11) | 0.217 | 0.73 (0.49, 1.10) | 0.130 | 0.73 (0.49, 1.10) | 0.138 | *Ref* |  | *Ref* |  | *Ref* |  |
| High (> €19,500) | 1.09 (0.79, 1.50) | 0.593 | 0.73 (0.47, 1.15) | 0.176 | 0.68 (0.43, 1.08) | 0.103 | 1.56 (0.95, 2.56) | 0.079 | 0.80 (0.40, 1.58) | 0.515 | 0.75 (0.38, 1.49) | 0.409 |
| **Chronic diseases (at least one)** | | | | | | | | | | | | |
| No | - | - | *Ref* |  | *Ref* |  | - | - | *Ref* |  | *Ref* |  |
| Yes | - | - | 1.27 (0.90, 1.80) | 0.170 | 1.25 (0.88, 1.78) | 0.203 | - | - | 1.91 (0.86, 4.23) | 0.112 | 1.63 (0.72, 3.68) | 0.238 |
| **Use of preventive healthcare services** | - | - | 0.97 (0.85, 1.11) | 0.655 | 0.98 (0.86, 1.11) | 0.730 | - | - | 0.73 (0.53, 1.00) | 0.052 | 0.75 (0.55, 1.03) | 0.079 |
| **Trust in official guidelines** | - | - | **4.26 (3.68, 4.93)** | **<.001** | **3.96 (3.41, 4.61)** | **<0.001** | - | - | **5.71 (3.88, 8.39)** | **<.001** | **5.38 (3.65, 7.95)** | **<0.001** |
| **Satisfaction with the healthcare system** | - | - | 1.12 (0.96, 1.31) | 0.155 | 1.13 (0.96, 1.33) | 0.129 | - | - | 1.14 (0.78, 1.68) | 0.493 | 1.13 (0.77, 1.66) | 0.543 |
| **Following doctor’s instructions** | - | - | 1.10 (0.92, 1.31) | 0.322 | 1.07 (0.90, 1.28) | 0.443 | - | - | 1.22 (0.83, 1.80) | 0.315 | 1.21 (0.82, 1.78) | 0.339 |
| **Vaccination knowledge score** | - | - | - | - | **1.11 (1.05, 1.18)** | **<0.001** | - | - | - | - | 1.15 (0.98, 1.35) | 0.098 |
| Abbreviations: OR, odds ratio; CI, Confidence interval; ^a^Model 1: Sociodemographic characteristics on vaccination status (Yes vs. No); ^b^Model 2: Sociodemographic characteristics, presence of chronic diseases and attitudes towards healthcare services on vaccination status (Yes vs. No); ^c^Model 3: Sociodemographic characteristics, presence of chronic diseases, attitudes towards healthcare services and vaccination knowledge score on vaccination status (Yes vs. No); *No observations; Bold values indicate statistically significant associations (p<0.05). | | | | | | | | | | | | |
